# Analysis of Cryopreservation Protocols and Their Harmful Effects on the Endothelial Integrity of Human Corneas

**DOI:** 10.3390/ijms222212564

**Published:** 2021-11-22

**Authors:** Silvia Rodríguez-Fernández, Marcelino Álvarez-Portela, Esther Rendal-Vázquez, María Piñeiro-Ramil, Clara Sanjurjo-Rodríguez, Rocío Castro-Viñuelas, Jacinto Sánchez-Ibáñez, Isaac Fuentes-Boquete, Silvia Díaz-Prado

**Affiliations:** 1 Grupo de Investigación en Terapia Celular e Medicina Rexenerativa, Departamento de Fisioterapia, Medicina e Ciencias Biomédicas, Facultade de Ciencias da Saúde, Universidade da Coruña (UDC), Campus de Oza, 15006 A Coruña, Spain; s.rodriguezf@udc.es (S.R.-F.); m.pramil@udc.es (M.P.-R.); clara.sanjurjo@udc.es (C.S.-R.); rocio.castro@udc.es (R.C.-V.); i.fuentes@udc.es (I.F.-B.); 2 Grupo de Investigación en Terapia Celular e Medicina Rexenerativa, Centro de Investigacións Científicas Avanzadas (CICA), Universidade da Coruña (UDC), 15071 A Coruña, Spain; 3 Grupo de Investigación en Terapia Celular e Medicina Rexenerativa, Instituto de Investigación Biomédica de A Coruña (INIBIC), Complexo Hospitalario Universitario A Coruña (CHUAC), Servizo Galego de Saúde (SERGAS), Universidade da Coruña (UDC), 15006 A Coruña, Spain; 4 Servizo de Oftalmoloxía, Complexo Hospitalario Universitario A Coruña (CHUAC), Servizo Galego de Saúde (SERGAS), 15002 A Coruña, Spain; marcelinoalvarezportela@gmail.com; 5 Unidade de Criobioloxía-Banco de Tecidos, Complexo Hospitalario Universitario A Coruña (CHUAC), Servizo Galego de Saúde (SERGAS), 15006 A Coruña, Spain; esther.rendal.vazquez@sergas.es (E.R.-V.); jacinto.sanchez.ibanez@sergas.es (J.S.-I.)

**Keywords:** corneal cryopreservation, cryoprotective agent, corneal endothelium, corneal storage, corneal transplantation, DMSO

## Abstract

Corneal cryopreservation can partially solve the worldwide concern regarding donor cornea shortage for keratoplasties. In this study, human corneas were cryopreserved using two standard cryopreservation protocols that are employed in the Tissue Bank of the Teresa Herrera Hospital (Spain) to store corneas for tectonic keratoplasties (TK protocol) and aortic valves (AV protocol), and two vitrification protocols, VS55 and DP6. Endothelial viability and general corneal state were evaluated to determine the protocol that provides the best results. The potential corneal cryopreservation protocol was studied in detail taking into consideration some cryopreservation-related variables and the endothelial integrity and stroma arrangement of the resulting cryopreserved corneas. TK corneas showed mostly viable endothelial cells, while the others showed few (AV) or none (DP6 and VS55). The corneal structure was well maintained in TK and AV corneas. TK corneas showed endothelial acellular areas surrounded by injured cells and a normal-like stromal fiber arrangement. Cryoprotectant solutions of the TK protocol presented an increasing osmolality and a physiological pH value. Cooling temperature rate of TK protocol was of 1 °C/min to −40 °C and 3 °C/min to −120 °C, and almost all of dimethyl sulfoxide left the tissue after washing. Future studies should be done changing cryopreservation-related variables of the TK protocol to store corneas of optical grade.

## 1. Introduction

The current corneal storage methods for keratoplasty only guarantee one month of optimal tissue preservation. Those are hypothermic and organ culture storage [1,2]. The storage time limitation and the increasing worldwide scarcity of donor corneas should encourage us to find a long-term alternative for viable corneal storage. This alternative would be provided by cryopreservation [2,3].

During the past decades, many corneal cryopreservation protocols were tested, particularly after the publication of the promising results of Capella et al. [4] and O’Neill et al. [5]. Based specially on the protocol of Capella et al., who used dimethyl sulfoxide (DMSO) as the main cryoprotective agent (CPA), several research groups analyzed some variables related to the corneal cryopreservation procedure and the corneal state after cryopreservation [6]. Afterwards, other cryopreservation parameters were tested, like different combinations and concentrations of CPAs in different vehicle solutions [7,8], different methods for CPA addition and removal and different cooling and warming rates, all to try to avoid cryoinjuries in corneas [9,10]. In that context, corneal vitrification protocols were designed [7,11,12,13]. This type of cryopreservation was considered the most appropriate if a viable tissue storage is desired, like corneas, cartilage, or aortic valves [14,15]. Theoretically, vitrification avoids ice formation inside cells and in tissue matrix using a high concentration of CPAs and superfast cooling and warming rates [16,17]. Standard cryopreservation only allows to control and minimizes ice crystal formation using a minor concentration of CPAs to avoid cytotoxicity when temperatures decrease or increase during the process [11,16,18,19].

In corneal cryopreservation, the evaluation of protocols is usually focused on the state of corneal endothelium. Endothelium is the main corneal layer responsible for maintaining stromal dehydration and, therefore, corneal transparency. This cell monolayer acts as a pump-leaky barrier that regulates the transport of solutes and water between aqueous humor and stroma [20]. As the endothelium has a limited capacity of regeneration [21], the extreme loss of endothelial cells (ECs) and/or of their functionality would be inadmissible if a viable cornea of optical grade is required. Because of this reason, the state of endothelium after cryopreservation is the best indicator of a successful corneal cryopreservation protocol [18].

Despite all attempts, any cryopreservation protocol for human corneas has not provided a tissue with all the endothelial qualities required to ensure a valid cornea [1]. Normally, corneas after cryopreservation showed a disrupted endothelial integrity [3,18,22], low EC viability, or an impaired endothelial function [23,24]. Furthermore, although human cryopreserved corneas have been used in penetrant keratoplasties, the high variability of clinical results pushed the cryopreservation technique to the background. Cryopreservation, as of now, is recommended only in emergencies for tectonic keratoplasty [25,26].

In this study, we evaluated the results of four cryopreservation protocols to store viable human corneas. Two of the protocols are standard cryopreservation procedures with the same controlled freezing temperature. Both are used in the Tissue Bank of the Teresa Herrera Hospital (Spain) to cryopreserve corneas for tectonic keratoplasties (TK protocol) and to cryopreserve aortic valves (AV protocol). The other two are experimental protocols of vitrification. VS55 was tested before with other small tissues that require viability [14,15], and DP6 is a version of VS55 without the CA formamide. We evaluated the endothelial viability immediately after thawing as well as the general corneal structure integrity. Then, we analyzed in detail the cryopreserved tissue that showed the best viability and histological results and the cryopreservation-related variables of the protocol. This way, we will have a starting point to improve the cryopreservation protocol to obtain a cryopreserved cornea of optical grade.

## 2. Results

### 2.1. Endothelial cell Viability of Cryopreserved Corneas

**Figure 1:**
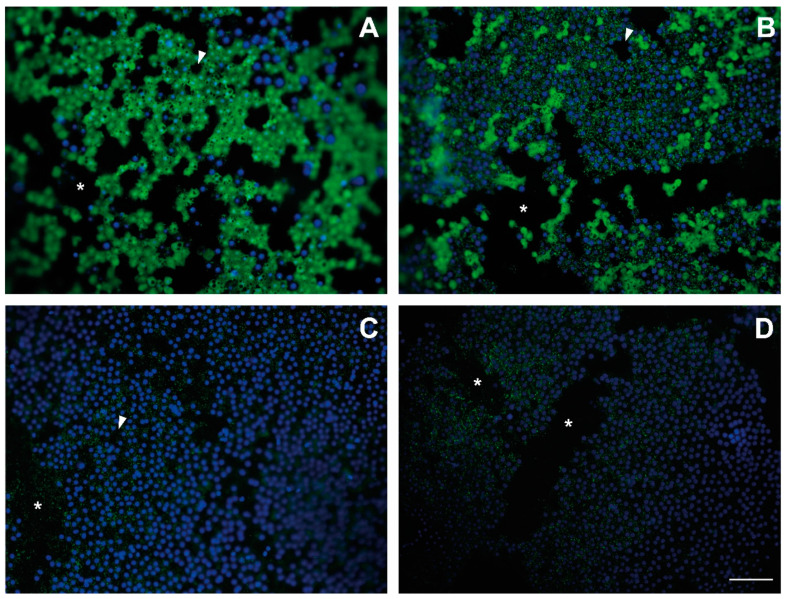
Pictures of corneal endothelia of thawed cryopreserved corneas after CPA removal and calcein AM assay. Endothelium of thawed cryopreserved cornea of (**A**) TK protocol, (**B**) AV protocol, (**C**) VS55 protocol, and (**D**) DP6 protocol. Nuclei of viable and non-viable cells are stained by Hoechst (blue). The cytoplasm of viable cells with intact cell membranes and enzymatic activity contains calcein AM (green), while the cytoplasm of non-viable cells with damaged cell membrane and/or non-enzymatic activity shows a green dotted cytoplasm or no signal of calcein AM; white arrows: small acellular areas; *: large strip-like acellular areas. Scale: 100 μm.

EC viability was analyzed qualitatively. The endothelia of TK-cryopreserved corneas showed mostly attached viable cells with intact cell membranes and enzymatic activity (Figure 1A), while in AV-cryopreserved corneas, endothelial cells remained attached but were mostly non-viable (Figure 1B). Some viable cells showed non-stained black vesicles on the cytoplasm. With respect to vitrification protocols, non-viable cells in the endothelia of VS55- and DP6-cryopreserved corneas were observed, although stained nuclei revealed that cells remained attached to Descemet’s membrane (Figure 1C,D).

In all cryopreserved endothelia, even in the fresh control (data not shown), the cell monolayer was disrupted, showing acellular areas on Descemet’s membrane and, therefore, decreasing the endothelial cell density. In cryopreserved corneas, those areas were numerous and of different shapes and sizes, for example, long strips or small spaces without any cell (Figure 1), while in the fresh control, there were very few small acellular areas that possibly indicated the loss of one cell on the highly viable endothelium.

### 2.2. General Corneal Structural Integrity

**Figure 2:**
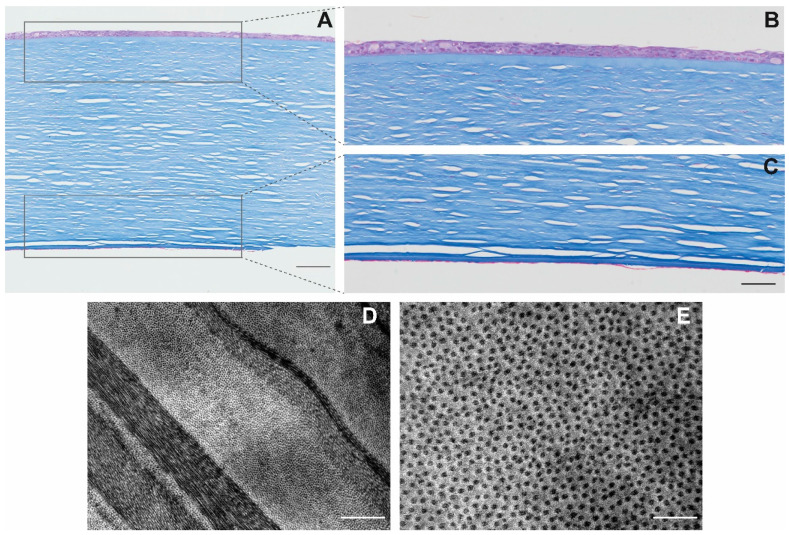
Transversal cuts of TK-cryopreserved corneas stained with Masson’s trichrome. All corneal layers are visible (**A**) (scale: 100 μm). Details of the epithelium (**B**) and the endothelium (**C**) (scale: 50 μm). TEM image of the banded pattern of collagen fibers in P1-cryopreserved corneas (**D**) (scale: 1 μm), with the details visible in a transversal cut of the collagen fibers (**E**) (scale: 200 nm).

Masson’s trichrome staining showed disrupted endothelia in all thawed cryopreserved corneas but in different ways. Only TK corneas and AV corneas showed cell debris but not a complete cell monolayer attached to Descemet’s membrane, with few intact ECs (Figure 2A,C). In DP6 and VS55 corneas, there was no endothelium in almost all cornea slides but some minimal cell debris at the periphery.

With respect to the stroma, a disruption in the collagen matrix was visible in the posterior (endothelial) part in TK-, DP6-, and VS55-cryopreserved corneas, being minimal in the anterior (epithelial) part, probably due to the quick uptake of water during the cryopreservation process. The stroma of TK- and AV-cryopreserved corneas were similar to the hypothermic-storage corneal control (not shown).

Epithelia of TK- and AV-cryopreserved corneas suffered flattening and desquamation, maintaining from two to three layers in the central part of the cornea (Figure 2A,B). In the case of TK cornea, epithelium remained attached in all extensions, while in the case of AV-cryopreserved cornea, a separation between endothelia and Bowman’s layers existed. The endothelia of VS55- and DP6-cryopreserved corneas were not visible in the central part of the corneas, but at the periphery, close to the trabecular meshwork.

As the TK protocol showed a higher number of viable cells and a more similar preserved cornea structure with respect to the control cornea, we discarded the other protocols for subsequent analysis.

### 2.3. Stromal Collagen Distribution of TK-Cryopreserved Corneas

**Figure 3:**
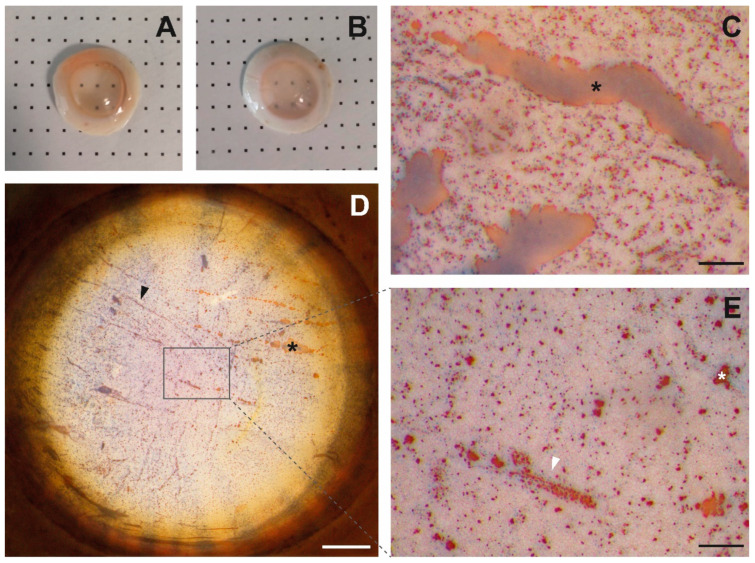
Vital stain in two TK-thawed cryopreserved corneas. The first cornea (**A**) shows a relative transparency that enables one to see the bold dots, while the second cornea (**B**) is more translucid and shows an endothelium with large strips, big amorphous spaces (black *), and a high amount of non-viable cells (blue nuclei) (**C**). The endothelium of a cornea (**A**) showing folds with non-viable cells on them (black arrow) (**D**) (scale: 1.5 mm), as well as small, acellular areas (white *) surrounded by non-viable cells (white arrow) (**E**) (scale: 200 μm).

TK-cryopreserved corneas presented a stroma with a regular collagen arrangement (Figure 2D), without the presence of possible breaks due to ice crystals. The distance among fibers in the two thawed corneas was 23.2 ± 7.7 nm and 27.1 ± 7.9 nm (Figure 2E), which was significatively superior to the interfibrillar distance in fresh cornea (19.4 ± 6.1 nm; *p* = 0.00). However, TK-cryopreserved corneas showed relative transparency (Figure 3A), with some exceptions (Figure 3B).

### 2.4. Endothelial Integrity of TK-Cryopreserved Corneas

Vital stain allowed observing of acellular areas in Descemet’s membrane stained in red in the endothelia of TK-cryopreserved corneas. The size and quantity of acellular areas varied among endothelia. These areas were usually localized at the periphery and/or in the central part of the endothelium, similar to large stripes (Figure 3C,D), big amorphous spaces (Figure 3C), or small spots (Figure 3E). The remaining attached cells frequently showed some blue-stained nuclei, indicating cell permeability (Figure 3C–E). In some cases, these cells were observed in all the extensions of the endothelium and in groups; however, they were mainly present surrounding large or small bare acellular areas (Figure 3E) or on Descemet’s membrane folds (Figure 3D).

### 2.5. Parameters of TK-Cryoprotectant Solutions

The osmolalities of TK cryoprotectant solutions (CSs) increased with the increasing of DMSO concentrations. The osmolalities of CS1-TK, CS2-TK, and CS3-TK were 639.0 ± 1.5, 1026.7 ± 4.2, and 1647.0 ± 7.2 mOsm/kg, respectively, the last one being hyperosmotic with respect to the albumin washing solution (274.0 ± 1.0 mOsm/kg; *p* = 0.01). The pH of all solutions was maintained at the physiologic level of 7.3 (21 °C).

### 2.6. Sample Cooling Profile of TK protocol

**Figure 4:**
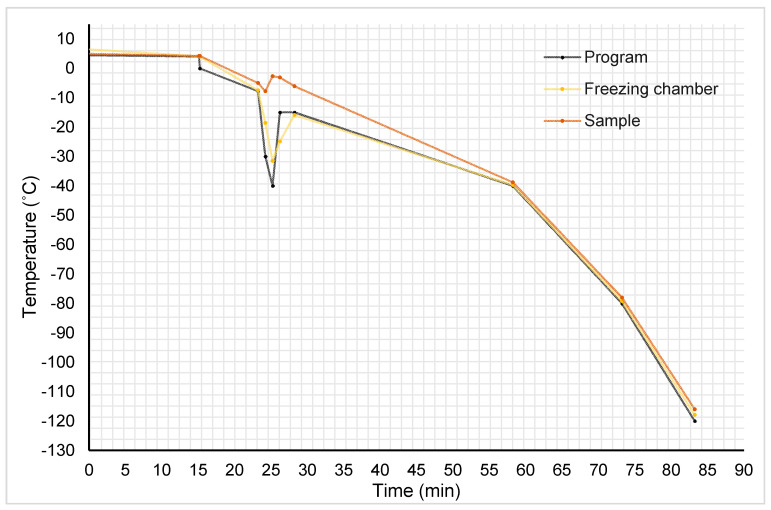
Graphic representation of temperature (*y*-axis) versus time (*x*-axis) during the cooling process of TK-cryopreserved corneas. In black, the temperature program; in yellow, the recorded temperature in the freezer chamber; in orange, the recorded temperature of samples.

The sample cooling profile of protocol TK is represented in Figure 4 and the variations in cooling rates in each segment of the programmed profile are displayed in Table 1. In brief, corneal samples were cooled slowly, reaching 4.2 °C. In that moment, the shock cooling forced ice nucleation with the consequent liberation of latent heat of fusion, which was partially counteracted by an abrupt decrease in the temperature chamber. The sample temperature was elevated from −7.9 to −2.7 °C in 27 s, achieving the equilibrium freezing point at −2.6 °C. A plateau at approximately −2.7 and −2.6 °C was maintained for 70.2 s, followed by a decrease in temperature. The velocity of the sample cooling rate increased progressively from –1.09 °C/min when the sample temperature was at −6 °C, to −3.80 °C/min when sample was at −78 °C.

### 2.7. DMSO Removal of TK-Cryopreserved Corneas

**Figure 5:**
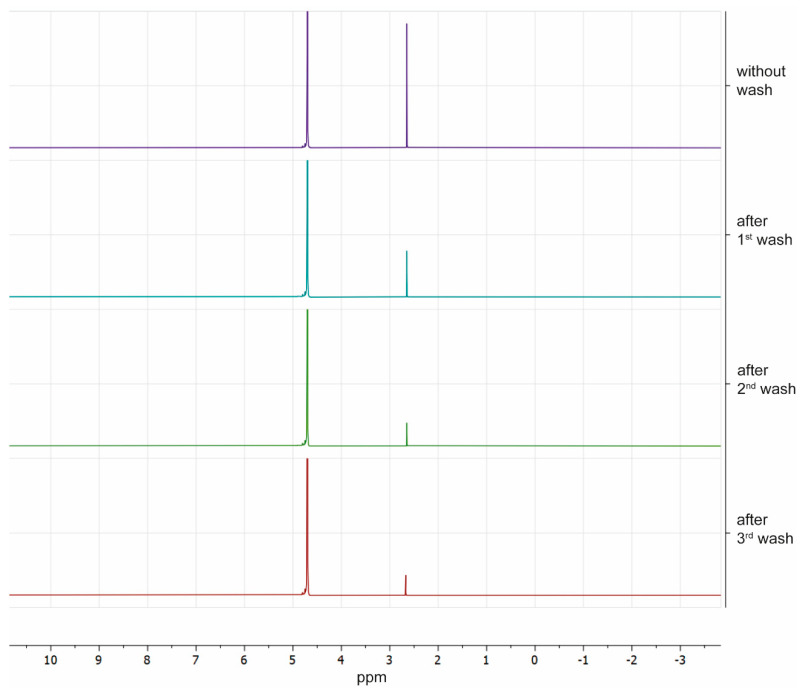
Spectra of proton nuclear magnetic resonance of each quarter of one TK-cryopreserved cornea after warming and washes. Peaks of water and DMSO are shown at 4.82 and 2.80 ppm, respectively. Areas of peaks are directly related with water and DMSO concentrations; ppm: particles per million.

The DMSO concentration in unwashed samples was 0.83 ± 0.08 M. A significant reduction of DMSO concentration was observed after the first wash (0.31 ± 0.05 M) and after the second wash (0.14 ± 0.01 M) but not after the third wash (0.10 ± 0.02 M) (Figure 5).

## 3. Discussion

In this study, we evaluated four corneal cryopreservation protocols attending specifically to corneal endothelial state, as endothelium is the major responsible for corneal transparency and the first indicator of a successful cryopreservation protocol [18].

Results show that all cryopreservation protocols are far from being usable in clinic, mainly due to endothelial disruption, which affects its functionality. However, TK-cryopreserved corneas are those that clearly offer a major amount of attached viable cells, so we recorded the cryopreservation-related variables of the TK-cryopreservation protocol. This protocol was selected as the potential protocol that could be improved to store valid functional corneas for keratoplasties.

Cryopreservation-related variables can explain partially the obtained endothelial viability and integrity results. These variables are the composition of CSs, the methods to add and remove CPAs, and the cooling and warming rates. Among cryoinjuries that cells can suffer, cell toxicity, osmotic stress, and mechanical injure by ice crystals are the most important.

CSs usually contain multimolar concentrations of CPAs that can be toxic for cells [13,17]. A low temperature and progressive contact with increasing or decreasing concentrations of CPAs in the addition or removal step should be considered in any cryopreservation protocol to reduce cytotoxicity [17].

Some CPAs, such as DMSO, proved to be low in cytotoxicity to ECs at low temperatures. Thus, DMSO was present in numerous standard cryopreservation and vitrification protocols from the beginning of corneal cryopreservation [3,4,18,27,28]. In TK and AV protocols, DMSO was added to corneas in cold CSs. Furthermore, the last CS of both protocols contains 2 M DMSO, a concentration that was demonstrated to be tolerated by ECs [8]. It is probable that cytotoxicity does not affect cells in the tested standard cryopreservation protocols, unlike vitrification protocols, where the elevated molarity of CPAs could be toxic for ECs. CPA addition in both vitrification protocols took place using a drop-by-drop technique, which could be too slow and in consequence, corneal cells would stay in contact with toxic concentrations of CPAs for a long time in a not-cold-enough CS. It was described that low temperatures or even subzero temperatures during CPA addition minimizes the cytotoxicity in a vitrification protocol [13].

For the four protocols, cell osmotic stress during addition and removal of CPAs could contribute to the loss of membrane integrity and/or of the enzymatic activity of ECs. Cells try to compensate any osmotic imbalance with medium. Abrupt osmolality changes can injure cells, causing extreme deturgescence or turgescence, or non-controlled ionic imbalance that can affect the functionality of the cellular system [17].

In our study, the non-stained vesicles in ECs after the calcein AM assay may be equivalent to those described by Wusteman et al. [28]. In that article, the authors hypothesized that the non-stained vesicles that they observed in the endothelia, just after DMSO addition, could be a cell response to osmotic and pH stress and would reflect the blebbing process that normally occurs before apoptosis.

Osmotic stress during cryopreservation can have several causes. The ionic composition of the vehicle solution of CSs is one of them. Some authors took this parameter into consideration, using ionic solutions that helped diminish the osmotic stress of EC in cold environments [10,13,27,28,29]. These solutions provided better results than a commercial tissue organ medium, similar to those that we employed in TK and AV protocols or those that other authors used in their corneal cryopreservation protocols [3,5,6,18,29].

The addition and removal steps are also critical at the cell osmotic level. It was reported that a gradual addition and removal of CPAs produces better results of endothelial cell viability as they help control cell osmotic stress [8,10,17]. Taking the example of the TK protocol, the differences among the increasing osmolalities of the consecutive CSs where corneas were embedded and the significative difference between the osmolalities of the third CS and the washing solution could not be osmotically well tolerated by ECs. For any of the four protocols, an increase in the number of CS solutions to reduce the osmolality leaps among them would lead to less osmotic stress in cells before freezing. In addition, for washing solutions, the same composition of CSs but with decreasing concentrations of CPAs would prevent a possible stress shock of ECs during CPA removal.

Toxic CPA removal is an important factor to avoid cell toxicity when cryopreserved corneas are at room temperature and if the cryopreserved cornea was transplanted, to prevent the toxic CPAs from passing to the receptor fluids or tissues. Thus, with NMR technology, we could measure the concentration of DMSO in TK-cryopreserved corneas and we observed that after washing, a concentration of only 0.1 M stayed in the tissue. Ideally, DMSO and any toxic CPAs should be reduced as much as possible before cell, tissue, or organ transplantation [30,31].

With respect to the cooling and warming rates, unadjusted speeds can lead to extracellular and intercellular ice formation that can mechanically injure tissue and cells. Uncontrolled intracellular ice formation could be responsible for mechanical injuries in the corneal endothelium [13,17]. The corneal endothelium is a monolayer of cells joined via GAP junctions. If intracellular ice is formed, these ice crystals can expand through GAP junctions, injure adjacent cells, and cause mechanical damages in cell membranes and the disruption of clusters of cells [32]. This would be able to justify the presence of the big acellular areas and the large acellular strips in all cryopreserved corneal endothelia because cells may be disrupted in groups and detached. In addition, ice crystals could be responsible for the loss of membrane integrity in the remaining ECs of VS55- and DP6- cryopreserved corneas. In these two cases, the non-viable ECs could be the result of devitrification, that is, the formation of ice crystals during warming if this is not done quick enough. Devitrification together with toxic CPA concentrations was described as one of the biggest challenges of vitrification [12,23]. To the best of our knowledge, only Armitage et al. [13] have succeeded in vitrifying rabbit corneas that retained functionality without devitrification.

It is mentioned that in standard cryopreservation, cooling rates of less than 1 °C/min and rates of 20 °C/min for warming can provide endothelial integrity and functionality [10]. Other cryopreservation protocols failed using temperature rates of 1 °C/min or superior, resulting in a corneal endothelium with acellular areas similar to those described in this study [18,33]. The cooling rate of TK protocol (and probably of AV protocol, as the cooling program was the same) was of approximately −1 °C/min to −40 °C and −3 °C/min to −120 °C. For this protocol, it would be necessary to design a cooling ramp that allows a slow decrease in the sample temperature to −80 °C.

Focusing on the general state of the cornea, a notable result that we observed in TK-cryopreserved corneas was the relative transparency, even when the interfibrillar distance in the transparent hypothermic-stored cornea was lower. Corneal transparency is related to the collagen fibers’ disposition in stromal lamellae and the interfibrillar distance among them [34]. The albumin of the wash solution could help to maintain the transparency, playing the role of an osmotic buffer to prevent stromal swelling, the way sucrose or mannitol did in other published corneal cryopreservation protocols [10,13,27,28]. Holes visualized in Masson’s trichrome may be the result of Masson’s own histological technique but also may be the result of corneal swelling changes during cryopreservation that separate lamellas.

Despite all this, we should consider that although cryopreserved corneal tissue is damaged during the cryopreservation process of cornea, injuries can start before cryopreservation, i.e., after donor death and during hypothermic storage.

After donor death, several anatomic and physiological changes occur in corneas. Napoli et al. detected in non-enucleated corneas, stromal thickness changes, separation of collagen lamellae, the flattened of epithelium due to the loss of tear films and water evaporation, or the failure of endothelium due to hypoxia [35,36,37]. This research group could detect these postmortem physiological changes in a non-invasive manner, using the portable optical coherence tomography (OCT) system. OCT is an useful technique to study the cornea surface and tear film [38,39,40] and to detect alterations caused by pathologies such as keratoconus or dry eye syndrome that can affect the corneal structure [41]. Corneas employed in this study were enucleated in the first 24 h postmortem, and although no corneal alterations were registered before enucleation, we should assume that some damage could be already present in the hypothermic stored samples.

Hypothermic conditions modify the corneal structure, and the corneas employed in this study were stored for days until being cryopreserved. Hypothermic storage usually creates folds in Descemet’s membrane [42]. These folds and the possible endothelium damage before enucleation could directly affect the endothelial results. The endothelium could be more susceptible to posterior cryoinjuries, mainly ECs that lie on Descemet’s folds, if not the whole endothelium. Hence, ECs would more easily detach from Descemet’s membrane or would turn into non-viable cells after a cryopreservation process.

A clear limitation of this study was the low number of samples and the interindividual variability, which is a common limitation in other studies of human corneal cryopreservation [18]. Another limitation of this study was the employed histological techniques, which only provides a first photograph of the cryopreserved corneas after thawing. After cryopreservation, it is necessary to perform experiments of corneal permeation to study the endothelial functionality [10,13,23,28] and to understand what can occur if it is transplanted. That way, we can see if the initially damaged or apparently intact ECs would be able to restore their functionality or directly suffer apoptosis and detach, causing stroma edema. In addition, and despite the current scarcity of donor corneas, it would be desirable to increment the number of samples of the study, homogenize the characteristics of samples, and make a complete study of corneas before enucleation and just before cryopreservation, using non-invasive techniques, such as OCT, and equipment such as the slip lamp and the specular microscope.

Definitively, none of the cryopreservation protocols were valid for being implemented in clinic. However, this study allows us to detect the drawbacks of the TK protocol. The cryopreservation-related variables of the current protocol will provide us a start point to improve it and to obtain a corneal cryopreservation protocol for storing valid corneas for keratoplasties.

## 4. Materials and Methods

### 4.1. Corneal Samples

The present study was approved by the Ethics Committee of Research from A Coruña-Ferrol, Spain (registration code: 2017/594). Cadaveric donor human corneas were obtained from corneas that were discarded by the Tissue Bank of Teresa Herrera Hospital (Galicia, Spain). We included all the corneas that were not valid for use in clinic except those that were discarded due to donor infection, with an ECD below 1500 cell/mm^2^, endothelial pathology, or alteration in the endothelium. The main reasons of corneal discard were arcus senilis, an ECD inferior to 2000 cell/mm^2^, infiltrates, leukoma, and the non-transplantation of a valid cornea after 7 days in hypothermic storage. Corneal samples were stored from 5 to 15 days in Eusol-C media (AlchimiA, Padova, Italia) at 4 °C until their use. The average donor age was 67 ± 6 years.

### 4.2. Cryoprotectant Solutions

Cryoprotectant solutions (CSs) of the TK protocol (CS-TK) consisted of a mixture of Medium 199 1X (M199; Gibco, Madrid, Spain), a perfusion solution of 20% human albumin (Albutein 20%; Grifols, Barcelona, Spain), and dimethyl sulfoxide (DMSO; Sigma, Madrid, Spain); for AV protocol, they consisted of M199 and DMSO; for VS55 protocol, they consisted of Euro-Collins 5X [15], propylene glycol (Sigma, Madrid, Spain), DMSO, formamide (Sigma, Madrid Spain), 4-(2-hydroxyethyl)-1-piperazineethanesulfonic acid (HEPES; Panreac, Barcelona, Spain), and distilled water; for DP6 protocol, they included Euro-Collins 5X, propylene glycol, DMSO, and HEPES. The concentrations of the components of the CSs are shown in Table 2. The CS were maintained at 4 °C for at least 1 h before use.

For each CS of TK protocol, the osmolality was measured in triplicate with a cryoscopic osmometer (Osmomat 030; Gonotec, Berlin, Germany) and the pH of each solution was determined using the pH-meter GLP21 (Crison, Castellón, Spain). Statistical analysis was performed with GraphPad Prisma software using a non-parametric test (Kruskal–Wallis test) to compare osmolalities among the different solutions, considering significant a *p*-value < 0.05. Results were expressed as the mean ± standard deviation.

### 4.3. Addition of Cryoprotectant Agents and the Cooling Process

Corneas were randomly used for the different cryopreservation protocols. Addition of CPAs to TK and AV protocols was done gradually. Corneas for TK protocol and corneas for AV protocol were transferred from the CS with a lower concentration of DMSO to the next CS with a higher concentration of DMSO, in intervals of 3 min. Finally, they were transferred into a precooled cryovial with the third CS of TK protocol (CS3-TK) or with the third CS of AV protocol (CS3-AV) and they were gently shaken on ice (4 °C) for 10 min. Immediately, they were introduced into the freezing chamber of the biological freezer CM-2010 (Carburos Medica, Barcelona, Spain) precooled at 4 °C. The cooling process was carried out following a temperature program, detailed in Table 3. Cryovials were stored in the gas phase of liquid nitrogen. For TK protocol, the sample cooling profile was studied using the report of the cryopreservation process generated by the biological freezer.

For VS55 and DP6 protocols, corneas were deposited directly in a precooled cryovial and respective cold CS were added drop-by-drop. Immediately, the cryovials were frozen in the liquid phase of a glass with isopentane precooled with liquid nitrogen. After 10 min in the cold liquid isopentane, the cryovials were transferred to the gas phase of liquid nitrogen for storage.

### 4.4. Warming Process and the Removal of Cryoprotectant Agents

For the warming process, cryovials from liquid nitrogen were maintained for 1 min at room temperature (RT), independently of the protocol used for freezing. Then, the cryovials were introduced in a water bath at 37 °C, without shaking, until complete thawing. For TK protocol, the corneas were immersed consecutively in three cold wash solutions of 20% albumin at 5 min intervals. For AV, VS55 and DP6 protocols, cryoprotectant media were eliminated using progressive dilutions (*v*/*v*) with cold saline buffer (Fisiológico B.Braun 0.9%; B. Braun Spain, Barcelona, Spain) (AV) and Euro-Collins 1X (VS55 and DP6), at 5 min intervals, until the cornea remained only in the washing solution. The thawed corneas were immediately processed for posterior assays.

### 4.5. Endothelial Cell Viability Assays

A calcein AM assay (LIVE/DEAD Cell Imaging Kit; Invitrogen, Barcelona, Spain) was performed on a peeled-off endothelial of two cryopreserved corneas from each protocol, following the manufacturer’s instructions of the kit and counterstaining with Hoechst (Sigma, Barcelona, Spain). Endothelia were visualized with the Olympus BX61 microscope (Olympus España S.A., Barcelona, Spain), coupled with the digital camera Olympus DP70 (Olympus España S.A., Barcelona, Spain). The open software FIJI [43] was used for image merging.

A vital stain with trypan blue and alizarine red was used to determine the endothelial integrity of 10 TK-cryopreserved corneas. Based on the protocol of Taylor et al. [44], thawed corneas were rinsed twice with phosphate buffer saline (PBS), stained with 0.2% trypan blue (Gibco, Madrid, Spain) for 90 s, rinsed twice with PBS, stained with 0.2% alizarin red (pH 4.2; Panreac, Barcelona, Spain) for 90 s, and rinsed twice before their pictures were taken with the camera Nikon digital sight DS-Fi2 coupled to the stereoscopy Nikon SM7 745T.

### 4.6. Measurement of the DMSO Concentration in Thawed Corneas

The DMSO concentration was measured in TK-cryopreserved corneas after they were thawed and after each step of the cryoprotectant removal. Proton (^1^H) nuclear magnetic resonance (NMR) spectroscopy was used to measure the DMSO concentration, as this molecule presents a characteristic peak in the NMR spectrum, as described before [9,27,28]. For measurement, three corneas were divided in four pieces after being thawed in the water bath. One-quarter of each cornea was submerged in a vial with deuterium oxide (D_2_O; Sigma, Madrid, Spain). The three remaining quarters were washed with the first albumin washing solution and after 5 min, one-quarter of each cornea was transferred to a vial with D_2_O, and the other two-quarters were transferred to the second albumin wash. This process was repeated with the remaining quarters of the cornea in the following washing steps. All pieces of the corneas were left in D_2_O at room temperature for 24 h to allow equilibration before they were frozen at −20 °C for storage.

To determinate the DMSO concentration, samples were thawed and the bathing D_2_O solution was transferred into a glass NMR tube for ^1^H NMR spectroscopy and analyzed with the Bruker AVANCE III HD 400.l spectrometer (Bruker, Billerica, MA, USA). Spectra were acquired after a strong pulse. All the spectra were Fourier transformed with the software MNova (Mestrelab Research) and automatically phased and integrals of each water and DMSO peaks were calculated to obtain the absolute value. The concentrations of DMSO were extrapolated using an experimental curve of the chemical relation between DMSO and water proton molar ratio for different concentrations of DMSO in milliQ water, as described before [9]. Statistical analysis was performed with GraphPad Prisma software using a non-parametric test (Kruskal–Wallis test with Bonferroni correction) to compare concentrations among the different groups, considering significant a *p*-value < 0.05. Results were expressed as the mean ± standard deviation.

### 4.7. Histomorphological Corneal Assessments

For each protocol, one thawed cornea was fixed in 4% formaldehyde and embedded in paraffin. Tissue sections were stained with Masson’s trichrome, and images were taken with the digital camera Olympus DP70 coupled to the Olympus BX61 microscope.

Two thawed TK-cryopreserved corneas and one fresh cornea were prepared for TEM. The central parts of the corneas were cut into small pieces and prefixed for 30 min at RT with 1.25% glutaraldehyde (Guimana, Valencia, Spain) and 0.1% paraformaldehyde (Thermo Fisher Scientific, Madrid, Spain) diluted in cacodylate buffer (1 M; pH 7.4), for better preservation of stromal collagen fibers [34]. After that, the tissue was fixed for 20 h in 2.5% glutaraldehyde, postfixed with 1% osmium tetroxide, followed by dehydration with increasing concentrations of acetone solutions, and embedded in resin. Ultrathin sections were processed and visualized with the JEOL JEM 1010 transmission electron microscope (JEOL, Tokyo, Japan).

A total of 100 stromal collagen fibers of the posterior stroma were used to measure the distance among the fibers using the open software FIJI. Statistical analysis was performed with GraphPad Prisma software using a parametric test (two-tailed *t*-test) to compare the mean of the interfibrillar distance of cryopreserved corneas and fresh cornea, considering significant a *p*-value < 0.05. Results were expressed as the mean ± standard deviation.

## Figures and Tables

**Table 1:** Sample cooling rate of each segment of the freezing program for the TK protocol. Although the temperature sample presents a fluctuation when nucleation is caused with a cooling shock, the temperature sample descends at a rate of approximately 1 °C/min to −38.8 °C and 3 °C/min to −116.6 °C.

	Cooling Rate (°C/min)	Duration (min)	Final Temperature (°C)
*Seg 1 + 2*	−0.05	15	4.2
*Seg 3*	−1.15	8	−5.0
*Seg 4*	−2.76	1	−7.7
*Seg 5*	+5.20	1	−2.7
*Seg 6*	−0.31	1	−3.0
*Seg 7*	−1.46	2	−6.0
*Seg 8*	−1.09	30	−38.8
*Seg 9*	−2.62	15	−78.0
*Seg 10*	−3.80	12	−116.6

**Table 2:** Component concentrations of cryoprotectant solutions (CSs) of the tested standard cryopreservation protocols (CPs) of corneas for tectonic keratoplasty (TK) and for aortic valves (AV) and experimental vitrification protocols (VP) VS55 and DP6. Concentrations are expressed in % (*v*/*v*), and penetrant cryoprotectants are also expressed in molarity.

	CP-TK			CP-AV		
	CS1-TK	CS2-TK	CS3-TK	CS1-AV	CS2-AV	CS3-AV
DMSO (*v*/*v*)	2% (0.3 M)	4% (0.6 M)	7% (1 M)	2% (0.3 M)	4% (0.6 M)	7% (1 M)
20% albumin (*v*/*v*)	25%	25%	25%	−	−	−
M199 1X ^a^ (*v*/*v*)	73%	71%	68%	98%	96%	93%
	**VP-VS55**			**VP-DP6**		
Propylene glycol (*v*/*v*)	16.25% (2.2 M)			22.04% (3.0 M)		
DMSO (*v*/*v*)	22.01% (3.1 M)			21.31% (3.0 M)		
Formamide (*v*/*v*)	12.31% (3.1 M)			−		
Euro-Collins 5X (*v*/*v*)	20.00%			56.65%		
Distilled water (*v*/*v*)	29.43%				−	
HEPES (g)	2.4 g			2.4 g		

**^a^** M199 1X: Medium 199 1X.

**Table 3:** Segments of the cooling profile used for corneal cryopreservation in TK protocol and AV protocol.

	Seg 1	Seg 2	Seg 3	Seg 4	Seg 5
Final Temperature (°C)	4.0	0.0	−7.8	−30.0	−40.0
Time (min)	15.0	0.1	8.0	1.0	1.0
	**Seg 6**	**Seg 7**	**Seg 8**	**Seg 9**	**Seg 10**
Final Temperature (°C)	−15.0	−15.0	−40.0	−80.0	−120.0
Time (min)	1.0	2.0	30.0	15.0	12.0

## References

[1] European Directorate for the Quality of Medicines and Healthcare (2017). Chapter 16. Ocular tissue. Guide to the Quality and Safety of Tissues and Cells for Human Application.

[2] Armitage W. (2011). Preservation of Human Cornea. Transfus. Med. Hemotherapy.

[3] Canals M., Garcia J., Potau J.M., Dalmases C., Costa-Vila J., Miralles A. (2000). Optimization of a method for the cryopreservation of rabbit corneas: Attempted application to human corneas. Cell Tissue Bank..

[4] Capella J.A., Kaufman H.E., Robbins J.E. (1965). Preservation of viable corneal tissue. Cryobiology.

[5] O’Neill P., Mueller F.O., Trevor-Roper P.D. (1967). On the preservation of corneae at −196 degrees C. for full-thickness homografts in man and dog. Br. J. Ophthalmol..

[6] Taylor M.J. (1986). Clinical cryobiology of tissues: Preservation of corneas. Cryobiology.

[7] Rich S.J., Armitage W.J. (1991). Corneal tolerance of vitrifiable concentrations of propane-1,2-diol. Cryobiology.

[8] Taylor M.J., Hunt C.J. (1989). Tolerance of corneas to multimolar dimethyl sulfoxide at 0 degrees C. Implications for cryopreservation. Investig. Ophthalmol. Vis. Sci..

[9] Walcerz D.B., Taylor M.J., Busza A.L. (1995). Determination of the kinetics of permeation of dimethyl sulfoxide in isolated corneas. Cell Biophys..

[10] Routledge C., Armitage W.J. (2003). Cryopreservation of cornea: A low cooling rate improves functional survival of endothelium after freezing and thawing. Cryobiology.

[11] Armitage W.J. (1989). Survival of corneal endothelium following exposure to a vitrification solution. Cryobiology.

[12] Bourne W.M., Nelson L.R. (1994). Human corneal studies with a vitrification solution containing dimethyl sulfoxide, formamide, and 1,2-propanediol. Cryobiology.

[13] Armitage W.J., Hall S.C., Routledge C. (2002). Recovery of endothelial function after vitrification of cornea at −110 °C. Investig. Ophthalmol. Vis. Sci..

[14] Brockbank K.G.M., Chen Z.Z., Song Y.C. (2010). Vitrification of porcine articular cartilage. Cryobiology.

[15] Brockbank K.G.M., Chen Z., Greene E.D., Campbell L.H. (2015). Vitrification of heart valve tissues. Methods Mol. Biol..

[16] Armitage W.J., Rich S.J. (1990). Vitrification of organized tissues. Cryobiology.

[17] Armitage W.J. (2009). Cryopreservation for Corneal Storage. Dev. Ophthalmol..

[18] Canals M., Costa-Vila J., Potau J.M., Merindano M.D., Ruano D. (1999). Morphological study of cryopreserved human corneal endothelium. Cells Tissues Organs.

[19] Rich S.J., Armitage W.J. (1990). Propane-1,2-diol as a potential component of a vitrification solution for corneas. Cryobiology.

[20] Bonanno J.A. (2012). Molecular mechanisms underlying the corneal endothelial pump. Exp. Eye Res..

[21] Joyce N.C. (2012). Proliferative capacity of corneal endothelial cells. Exp. Eye Res..

[22] Halberstadt M., Böhnke M., Athmann S., Hagenah M. (2003). Cryopreservation of Human Donor Corneas with Dextran. Investig. Ophthalmol. Vis. Sci..

[23] Wusteman M.C., Simmonds J., Vaughan D., Pegg D.E. (2008). Vitrification of Rabbit Tissues with Propylene Glycol and Trehalose. Cryobiology.

[24] Hagenah M., Böhnke M. (1993). Corneal cryopreservation with chondroitin surfate. Cryobiology.

[25] Brunette I., Le François M., Tremblay M.C., Guertin M.C. (2001). Corneal Transplant Tolerance of Cryopreservation. Cornea.

[26] Ohno K., Nelson L.R., Mitooka K., Bourne W.M. (2002). Transplantation of cryopreserved human corneas in a xenograft model. Cryobiology.

[27] Wusteman M.C., Armitage J.W., Wang L.H., Busza A.L., Pegg D.E. (1999). Cryopreservation studies with porcine corneas. Curr. Eye Res..

[28] Wusteman M.C., Boylan S., Pegg D.E. (1997). Cryopreservation of rabbit corneas in dimethyl sulfoxide. Investig. Ophthalmol. Vis. Sci..

[29] Taylor M.J., Hunt C.J. (1985). A new preservation solution for storage of corneas at low temperatures. Curr. Eye Res..

[30] Brockbank K.G. (2016). Removal of potentially cytotoxic DMSO from cell therapy cryopreservation formulations. MOJ Cell Sci. Rep..

[31] Díaz Rodríguez R., Van Hoeck B., De Gelas S., Blancke F., Ngakam R., Bogaerts K., Jashari R. (2017). Determination of residual dimethylsulfoxide in cryopreserved cardiovascular allografts. Cell Tissue Bank..

[32] Elliott J.A.W. (2013). Intracellular ice formation: The enigmatic role of cell-cell junctions. Biophys. J..

[33] Madden P.W., Taylor M.J., Hunt C.J., Pegg D.E. (1993). The Effect of Polyvinylpyrrolidone and the Cooling Rate during Corneal Cryopreservation. Cryobiology.

[34] Müller L.J., Pels E., Schurmans L.R.H.M., Vrensen G.F.J.M. (2004). A new three-dimensional model of the organization of proteoglycans and collagen fibrils in the human corneal stroma. Exp. Eye Res..

[35] Nioi M., Napoli P.E., Demontis R., Locci E., Fossarello M., D’Aloja E. (2018). Morphological analysis of corneal findings modifications after death: A preliminary OCT study on an animal model. Exp. Eye Res..

[36] Napoli P.E., Nioi M., Gabiati L., Laurenzo M., De-Giorgio F., Scorcia V., Grassi S., D’Aloja E., Fossarello M. (2020). Repeatability and reproducibility of post-mortem central corneal thickness measurements using a portable optical coherence tomography system in humans: A prospective multicenter study. Sci. Rep..

[37] Napoli P.E., Nioi M., d’Aloja E., Fossarello M. (2016). Post-Mortem Corneal Thickness Measurements with a Portable Optical Coherence Tomography System: A Reliability Study. Sci. Rep..

[38] Napoli P.E., Coronella F., Satta G.M., Galantuomo M.S., Fossarello M., Huang J. (2014). Evaluation of the adhesive properties of the cornea by means of optical coherence tomography in patients with meibomian gland dysfunction and lacrimal tear deficiency. PLoS ONE.

[39] Napoli P.E., Coronella F., Satta G.M., Fossarello M. (2014). A novel technique of contrast-Enhanced optical coherence tomography imaging in evaluation of clearance of lipids in human tears. PLoS ONE.

[40] Di Marino M., Conigliaro P., Aiello F., Valeri C., Giannini C., Mancino R., Modica S., Nucci C., Perricone R., Cesareo M. (2021). Combined Low-Level Light Therapy and Intense Pulsed Light Therapy for the Treatment of Dry Eye in Patients with Sjögren’s Syndrome. J. Ophthalmol..

[41] Napoli P.E., Nioi M., D’Aloja E., Loy F., Fossarello M. (2020). The architecture of corneal stromal striae on optical coherence tomography and histology in an animal model and in humans. Sci. Rep..

[42] Tran K.D., Clover J., Ansin A., Stoeger C.G., Terry M.A. (2017). Rapid warming of donor corneas is safe and improves specular image quality. Cornea.

[43] Schindelin J., Arganda-Carreras I., Frise E., Kaynig V., Longair M., Pietzsch T., Preibisch S., Rueden C., Saalfeld S., Schmid B. (2012). Fiji: An open-source platform for biological-image analysis. Nat. Methods.

[44] Taylor M.J., Hunt C.J. (1981). Dual staining of corneal endothelium with trypan blue and alizarin red S: Importance of pH for the dye-lake reaction. Br. J. Ophthalmol..

